# *In vivo* laser speckle contrast imaging of 4-aminopyridine- or pentylenetetrazole-induced seizures

**DOI:** 10.1063/5.0158791

**Published:** 2023-09-28

**Authors:** Yuhling Wang, Vassiliy Tsytsarev, Lun-De Liao

**Affiliations:** 1 Institute of Biomedical Engineering and Nanomedicine, National Health Research Institutes, No. 35, Keyan Rd., Zhunan Township, Miaoli County 350, Taiwan; 2 Department of Electrical Engineering, National United University, No. 2, Lien Da, Nan Shih Li, Miaoli City 36063, Taiwan; 3 Department of Anatomy and Neurobiology, University of Maryland School of Medicine, 20 Penn Street, HSF-2, Baltimore, Maryland 21201, USA

## Abstract

Clinical and preclinical studies on epileptic seizures are closely linked to the study of neurovascular coupling. Obtaining reliable information about cerebral blood flow (CBF) in the area of epileptic activity through minimally invasive techniques is crucial for research in this field. In our studies, we used laser speckle contrast imaging (LSCI) to gather information about the local blood circulation in the area of epileptic activity. We used two models of epileptic seizures: one based on 4-aminopyridine (4-AP) and another based on pentylenetetrazole (PTZ). We verified the duration of an epileptic seizure using electrocorticography (ECoG). We applied the antiepileptic drug topiramate (TPM) to both models, but its effect was different in each case. However, in both models, TPM had an effect on neurovascular coupling in the area of epileptic activity, as shown by both LSCI and ECoG data. We demonstrated that TPM significantly reduced the amplitude of 4-AP-induced epileptic seizures (4-AP+TPM: 0.61 ± 0.13 mV vs 4-AP: 1.08 ± 0.19 mV; *p* < 0.05), and it also reduced gamma power in ECoG in PTZ-induced epileptic seizures (PTZ+TPM: 38.5% ± 11.9% of the peak value vs PTZ: 59.2% ± 3.0% of peak value; *p* < 0.05). We also captured the pattern of CBF changes during focal epileptic seizures induced by 4-AP. Our data confirm that the system of simultaneous cortical LSCI and registration of ECoG makes it possible to evaluate the effectiveness of pharmacological agents in various types of epileptic seizures in *in vivo* models and provides spatial and temporal information on the process of ictogenesis.

## I. INTRODUCTION

Epilepsy is a serious brain disorder that affects 1% of the population worldwide.^1^ Pharmacotherapy is a highly effective way to treat epilepsy, but unfortunately, up to 20% of cases of epilepsy respond poorly or not at all to treatment with antiepileptic drugs. In such cases, other methods of treatment are used, such as surgical removal of the epileptic focus or deep brain stimulation (DBS). While the surgical outcome for temporal lobe epilepsy is extremely successful, with cure rates approaching 70%–80%, extratemporal neocortical seizures have a poor cure rate, ranging from 25% to 40%, and a high recurrence rate. Surgical therapy for extratemporal neocortical seizures can be unsuccessful when resection is incomplete, which results from incomplete mapping of the epileptic foci.^2^

Neocortical seizures pose a particular challenge in defining the region of epileptogenesis, which is critical for surgical resection. The population of neurons involved changes over time, and discrete neuronal networks may also be involved. The relationship between interictal events and ictal onset zones is poorly understood. Although the current “gold standard” is to use electrophysiological recording methods, including electroencephalography (EEG), there are still limitations when using these methods to define the region of epilepsy. Sampling limitations and volume conduction prevent high accuracy of spatial localization of the epilepsy network. Subdural grids and strip electrodes can be used to improve the definition of the boundary but are invasive and not completely risk free. The placement of these electrodes to cover the complete epileptogenic network remains a constant challenge. While recent developments in the utility of dipole modeling in scalp EEG and EEG source imaging are promising for improving the current gold standard EEG-based methods, there remains a need for developing new techniques to map the epilepsy circuitry in a noninvasive manner with high spatial and temporal resolution.^3^ The usage of voltage-sensitive dyes optical imaging (VSDi) combined with allows direct visualization of neuronal activity with higher spatial resolution than EEG-based methods. However, an imaging method that does not require an injection of a contrast agent is preferred. One alternative is to measure neuronal activity indirectly. Increased neuronal metabolism induces vasomotor responses and hemodynamic changes that can be measured in order to map epilepsy. The drawback of mapping neurovascular function is that the increase in cerebral blood flow (CBF) occurs at least hundreds of milliseconds after neuronal activity increases, which limits temporal resolution. Mapping epilepsy with high spatial and temporal resolution is highly important for both fundamental research and the clinical management of epilepsy; however, the question of how to visualize neurovascular activity in epileptic seizures remains unanswered.^4^

The localization of epileptic foci is commonly performed by functional magnetic resonance imaging (fMRI).^5,6^ However, changes in local blood flow or oxygenation typically do not occur during the interictal period (i.e., the period between seizures). Thus, fMRI application in epileptic research is very limited. Positron emission tomography (PET) is a noninvasive nuclear imaging method based on the detection of pairs of gamma quants emitted by a positron-emitting radionuclide. In some cases, epileptogenic parts of the brain demonstrate reduced glucose uptake (hypometabolism) during PET imaging. However, high cost, technical difficulties, and relatively low spatial and temporal resolution limit the use of PET in epileptic network mapping.^1,7^ Near-infrared spectroscopy (NIRS) is not only an optical technique used to analyze real-time changes in human CBF and oxygenation but also very appropriate for use in small animals (e.g., animal models) due to the reduced thickness of the skull.^8^ Tissue oxygenation and hemodynamic changes can be monitored continuously both during the interictal period and during seizures. However, it has much worse spatial resolution than human fMRI. Therefore, none of these technologies can visualize epileptic activity in the brain as a function of neurovascular activity or dynamics.

The imaging platform we developed can help to address this issue, at least in an animal model of epilepsy. As we showed in our previous publications regarding the use of laser speckle contrast imaging (LSCI) and functional photoacoustic microscopy (fPAM),^9–12^ we can visualize neurovascular activity in the brain of a small animal with a spatial resolution of a few tens of micrometers and a temporal resolution of up to 5 ms.^13^ The application of this imaging technology to study epileptic seizures can provide high temporal resolution of 2D structures in epileptic seizures. Comparing data obtained from untreated and treated experimental animals will allow us to draw conclusions about the nature of epileptic activity and its origin, spread, and termination in the brain.

Here, we used a rat model of epileptic seizures chemically induced by 4-aminopyridine (4-AP)^14^ or pentylenetetrazole (PTZ). Both of these *in vivo* models of epileptic seizures (i.e., those induced by 4-AP and PTZ) allow us to investigate the dynamics of the induction, maintenance, and spread of seizure discharges in the cortex. These models have been extensively studied using different optical and electrophysiological methods. Epileptic seizures are usually accompanied by a local increase in CBF to the epileptic focus, but the relationship between pathologically synchronized neuronal activity and local blood flow remains unclear. In the present study, we demonstrate for the first time 2D mapping of epilepsy *in vivo* via neurovascular activity with good spatial and temporal resolution in an animal model frequently used in epilepsy studies. We were able to characterize the induced epileptic seizures using our developed electrocorticography (ECoG)-LSCI system. The goal of this study was to expand previous findings regarding our ECoG-LSCI system by incorporating seizure treatment with the antiepileptic drug topiramate (TPM) to test its ability to evaluate treatment efficacy. Since the 4-AP model of epileptic seizures is induced by the blockade of K^+^ channels,^15^ additional models with different mechanisms (i.e., the PTZ model) are also necessary for adequate testing of seizure treatments. The PTZ model of epileptic seizures is induced by the inhibition of GABA_A_ receptors.^16^ Thus, our next goal was to use our ECoG-LSCI system to assess the efficacy of TPM treatment in the 4-AP and PTZ models of epileptic seizures. Our system allows us to characterize and quantify different aspects of the seizure models (i.e., 4-AP and PTZ) and provides a basis for evaluating treatment efficacy.

## II. RESULTS

### A. Characterization of 4-AP-induced epileptic seizures

One of the well-established *in vitro* and *in vivo* models of epileptic seizures is the 4-AP model. Previous studies have shown that this model is capable of recapitulating the antiepileptic efficacy of various antiepileptic drugs (AEDs), such as TPM.^14,17^ Many types of epilepsy have been linked to the pathology of potassium channels. Some mutations in potassium channels lead to hyperexcitability of neurons. Similarly, the potassium channel inhibitor 4-AP causes hyperexcitability and the development of seizure-like discharges.^16^ Thus, it is highly likely that 4-AP increases the excitability of both inhibitory and excitatory neurons and that inhibitory neurons play a key role in the development of seizure-like activity.^16^

The PTZ model is based on completely different principles and induces seizures by blockade of GABA_A_ receptors. Therefore, it is logical that PTZ-induced seizures would be sensitive to drugs that act on GABA_A._^16^ High-dose systemic administration of PTZ induces an acute seizure, whereas sequential injections of a subthreshold dose have been used for the development of chemical kindling. PTZ-based methods are widely applicable in epileptic studies, including screening of antiepileptic drugs.^18^

**FIG. 1.**
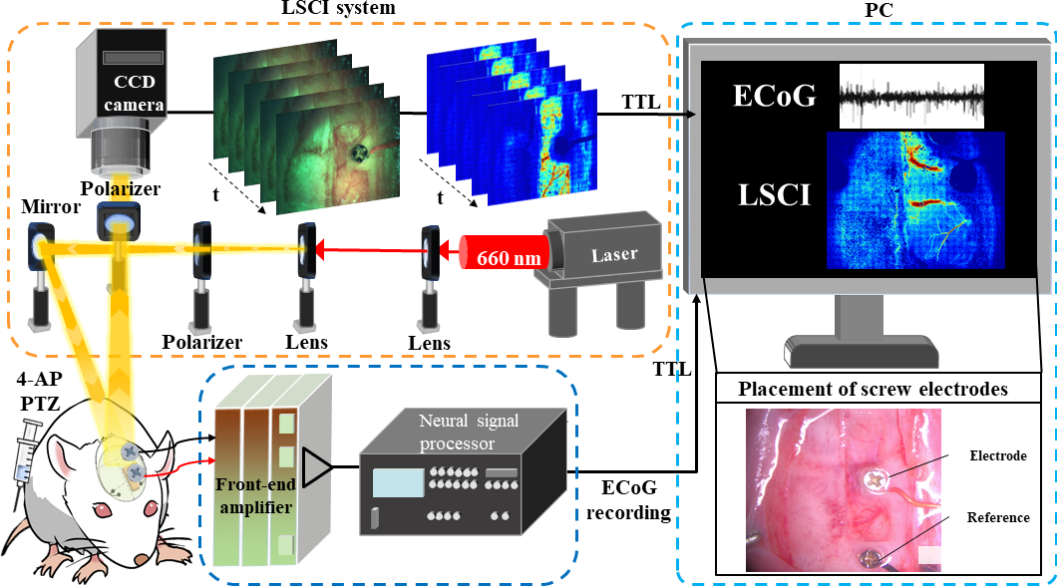
Schematic of the ECoG-LSCI system for monitoring epileptic seizures. Speckle patterns generated by laser light scattered and reflected from the brain surface were captured using a CCD camera and analyzed to monitor changes in CBF during seizures. ECoG signals from two epidural electrodes were simultaneously captured to examine the relationship between CBF and bioelectrical signals.

**FIG. 2.**
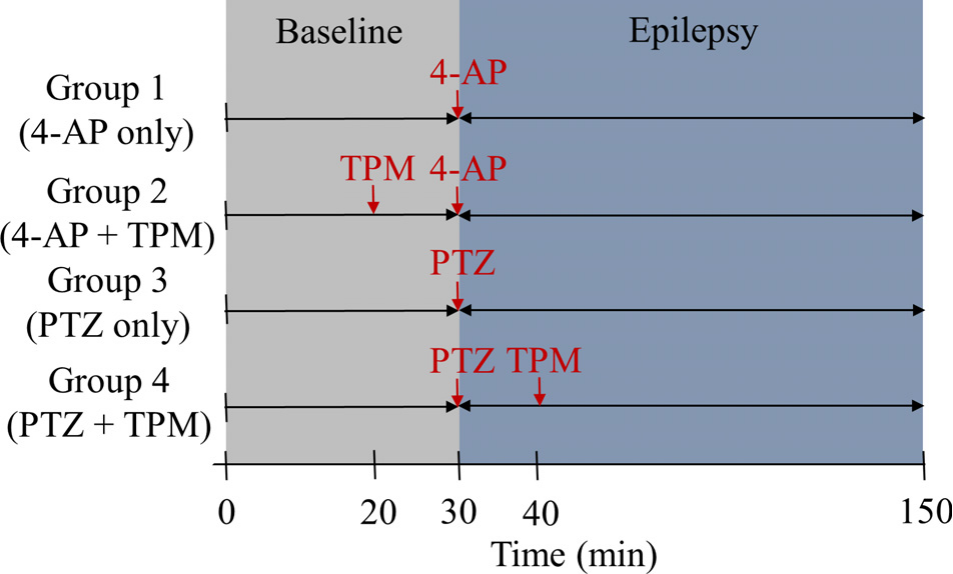
Schematic of the data collection sequence using the ECoG-LSCI system. Baseline values were recorded 30 min before the induction of epileptic seizures. Immediately after injection with 4-AP or PTZ, data were collected to monitor the induced epileptic seizures. Groups 1 and 3 are the untreated control groups with either 4-AP or PTZ seizure induction, while groups 2 and 4 are the treatment groups (with TPM injected i.p. 10 min before or after seizure induction).

**FIG. 3.**
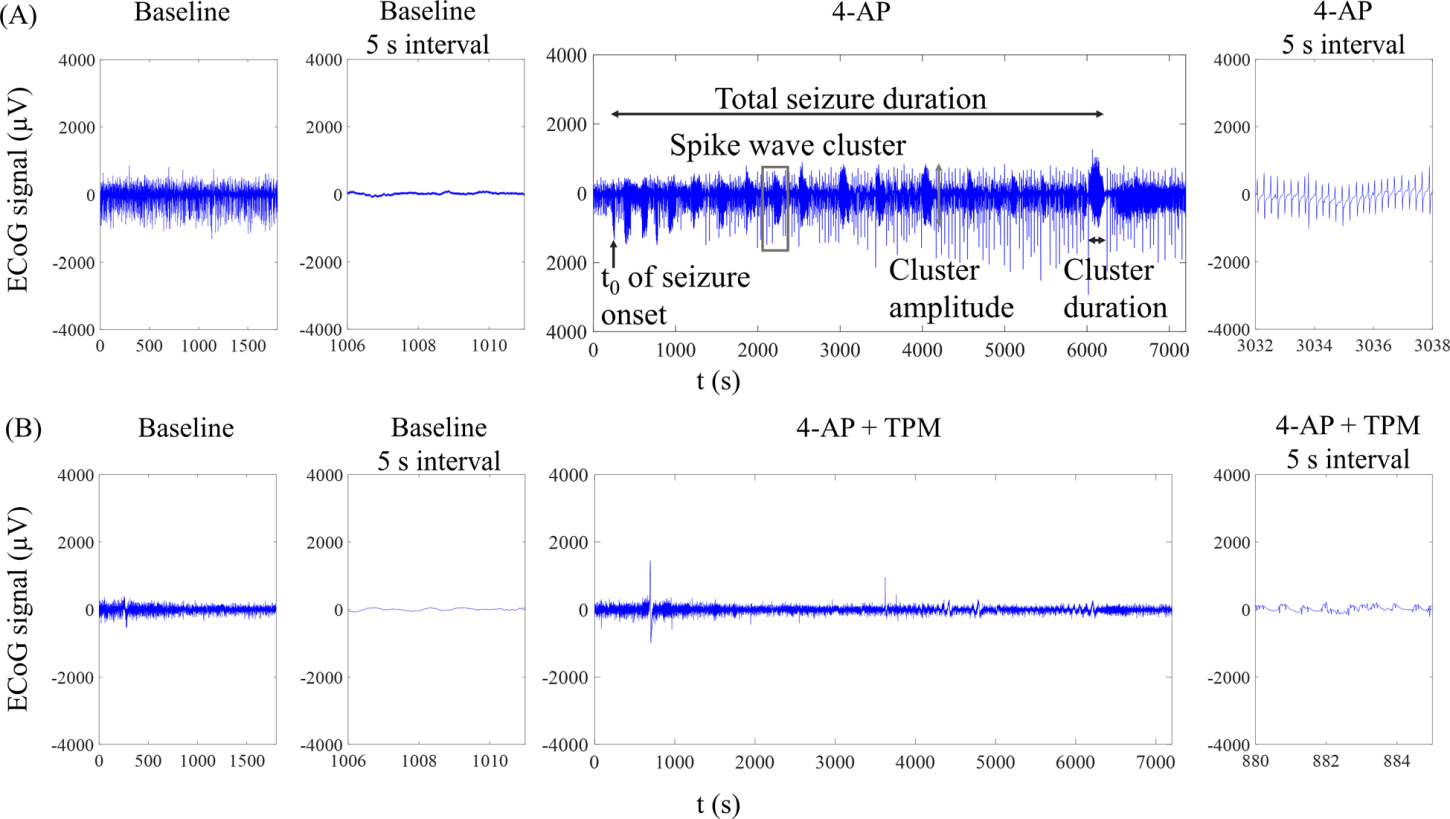
Representative ECoG data after 4-AP seizure induction. (a) Representative data from 4-AP-induced seizures. From left to right are the 30 min baseline signal, a 5 s interval of the baseline signal, the signal after 4-AP injection with a schematic of the quantification, and a 5 s interval of the 4-AP-induced seizure where spike waves are evident. (b) Representative data from a 4-AP+TPM rat. From left to right are the 30 min baseline signal; a 5 s interval of the baseline signal; the signal after 4-AP injection; and a 5 s interval of the 4-AP-induced seizure where spike waves can be observed, although they are less evident than those in an untreated seizure.

A schematic of the ECoG-LSCI system used to measure neural activity and cerebral blood flow is shown in Fig. 1. The data collection sequence during seizure induction is shown in Fig. 2. Representative ECoG data collected from 4-AP-induced seizures are shown in Fig. 3. The ECoG pattern of 4-AP-induced seizures without TPM treatment [Fig. 3(a)] was used to identify characteristics to monitor. No patterns were seen in the baseline signal, even when viewing at 5 s intervals. After injection of 4-AP, periods of high-frequency signals were observed in the recorded ECoG signal, which we defined as spike wave clusters. The spike waves can be seen more clearly when viewing the signal within a cluster at a 5 s interval. For data analysis, we quantified the total number of clusters detected during the 2 h of recording, the average duration of individual clusters, the maximum amplitude of clusters, the time of seizure onset (t_0_), and the total seizure duration (start of the first cluster to end of the last cluster).

The ECoG pattern of 4-AP-induced seizures with TPM treatment is shown in Fig. 3(b). Compared to untreated rats, seizure events were less evident unless examined at the 5 s timescale. Spike waves could be seen in the 5 s interval, although at a smaller amplitude. The average cluster amplitudes were smaller in the 4-AP+TPM rat than in the 4-AP rat (0.49 ± 0.06 mV vs 1.24 ± 0.14 mV).

### B. Characterization of PTZ-induced epileptic seizures

**FIG. 4.**
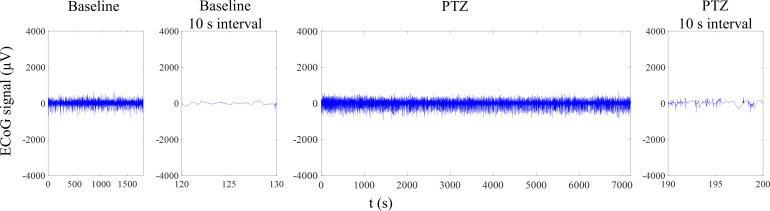
Representative ECoG data after PTZ seizure induction. From left to right are the 30 min baseline signal, a 10 s interval of the baseline signal, signal after PTZ injection, and a 10 s interval of the PTZ-induced seizure where spike waves are observed. Unlike 4-AP-induced seizures, clusters of spike waves are not evident.

Representative ECoG data collected from PTZ-induced seizures are shown in Fig. 4. In contrast to 4-AP-induced seizures, PTZ-induced seizures showed no clear delineation of spike waves into clusters. Spike waves were evident only when the data were viewed at 10 s intervals. Because of this, spectral-band power analysis was used.

### C. Comparison of 4-AP-induced epileptic seizures in untreated rats vs TPM-treated rats

**FIG. 5.**
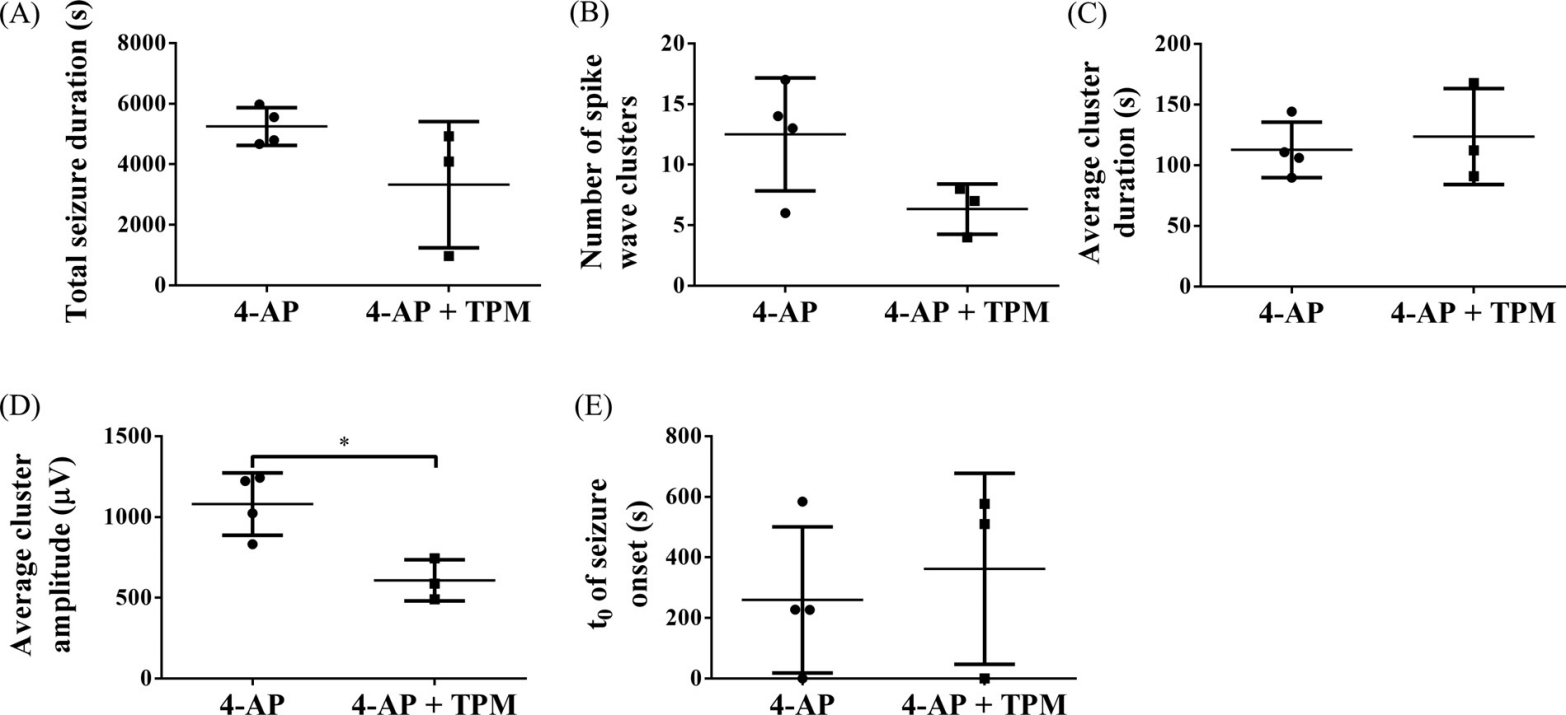
Comparison of induced epileptic seizures in 4-AP vs 4-AP+TPM rats. (a) Total seizure duration. (b) Number of spike wave clusters. (c) Average duration of spike wave clusters. (d) The average amplitude of spike wave clusters was significantly decreased in TPM-treated rats (unpaired *t* test with Welch's correction; ^*^p < 0.05; 4-AP: n = 4; 4-AP+TPM: n = 3). (e) Time of seizure onset (t_0_).

After establishing our rat model of 4-AP-induced epileptic seizures, we treated some rats with the antiepileptic drug TPM and compared the results to those of untreated rats. The results are shown in Fig. 5. The average cluster amplitude [4-AP+TPM: 0.61 ± 0.13 mV vs 4-AP: 1.08 ± 0.19 mV; Fig. 5(d)] was significantly lower (unpaired *t* test with Welch's correction; *p < 0.05; 4-AP: n = 4; 4-AP+TPM: n = 3) in 4-AP+TPM rats than in 4-AP rats. The total seizure duration [4-AP+TPM: 3.33 ± 2.08 × 10^3^ s vs 4-AP: 5.25 ± 0.62 × 10^3^ s; Fig. 5(a)], number of spike wave clusters [4-AP+TPM: 6 ± 2 vs 4-AP: 13 ± 5; Fig. 5(b)], and average cluster duration [4-AP+TPM: 1.24 ± 0.40 × 10^2^ s vs 4-AP: 1.13 ± 0.23 × 10^2^ s; Fig. 5(c)] were lower in 4-AP+TPM rats, but this difference was not significant. The time of seizure onset [4-AP+TPM: 3.62 ± 3.16 × 10^2^ s vs 4-AP: 2.60 ± 2.41 × 10^2^ s; Fig. 5(e)] was similar in 4-AP rats and 4-AP+TPM rats.

### D. LSCI characterization of 4-AP-induced epileptic seizures

**FIG. 6.**
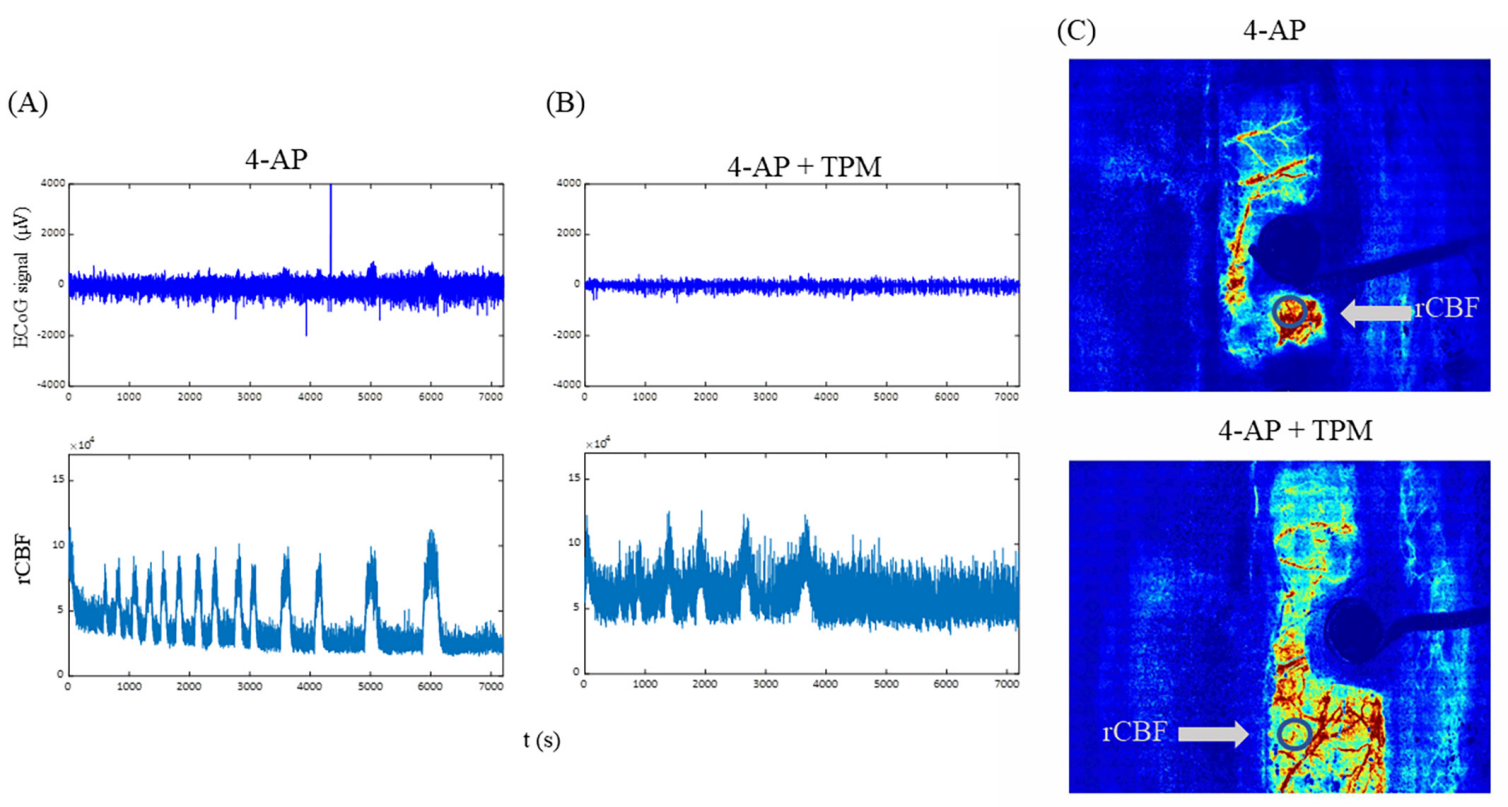
LSCI data collected from rats with 4-AP-induced epileptic seizures. (a) ECoG signals (top panel) and change in rCBF (bottom panel) of an untreated rat. Peaks in rCBF correspond to clusters in the ECoG signal, indicating that changes in blood flow occur during 4-AP-induced epileptic seizures. (b) Plots of ECoG signal (top panel) and rCBF (bottom panel) in a 4-AP+TPM rat. (C) LSCI images of cortical blood flow obtained after seizure induction from the rats in (a) and (b). The ROIs used to generate the rCBF plots are circled.

LSCI data recorded after 4-AP injection are shown in Fig. 6. LSCI uses speckles caused by interference between light scattered by moving RBCs in the tissue to create a representation of the blood flow distribution, as shown in Fig. 6(c). Representative ECoG and rCBF data from 4-AP-induced epileptic seizures are shown in Fig. 6(a). The peaks in the rCBF plot [Fig. 6(a), bottom] correspond to the seizure events detected using ECoG [Fig. 6(a), top], indicating that strong changes in CBF occur during seizures induced by 4-AP. Representative ECoG and rCBF data from 4-AP-induced epileptic seizures treated with TPM are shown in Fig. 6(b). Seizure is more evident in the rCBF plot compared to the ECoG signal in the TPM-treated animal. The regions of interest (ROIs) used to generate the rCBF plots in Figs. 6(a) and 6(b) are shown in Fig. 6(c). Videos of the change in rCBF can be seen in Movies 1 and 2.

### E. Comparison of PTZ-induced epileptic seizures in untreated rats vs TPM-treated rats

**FIG. 7.**
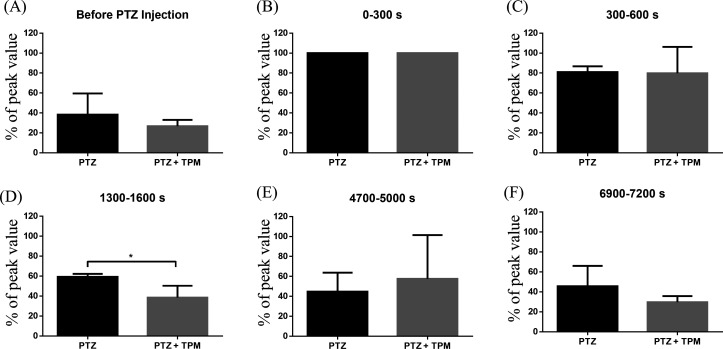
Spectral-band power analysis of PTZ-induced epilepsy. Gamma power was calculated for PTZ+TPM (n = 4) and PTZ (n = 3) rats and graphed as % of peak value, which occurred at 0–300 s. Gamma power values from six different 300 s intervals were compared. (a) Gamma power before PTZ injection. (b) Gamma power immediately after PTZ injection. (c) Gamma power after PTZ injection but before TPM injection. (d)–(f) Gamma power at three different time points after TPM injection. Gamma power was significantly reduced (unpaired t test with Welch's correction; *p < 0.05; PTZ: n = 3; PTZ+TPM: n = 4) in TPM-treated rats at the 1300–1600-s interval.

Since PTZ-induced seizures differed from 4-AP-induced seizures and did not present with visible clusters of spike waves, spectral-band power analysis was used (Fig. 7). At baseline [Fig. 7(a)], gamma power was low (PTZ+TPM: 26.8% ± 6.3% of the peak value vs PTZ: 38.5% ± 21.1% of the peak value). After PTZ injection at 0 s, gamma power increased to a peak value [Fig. 7(b)] and then gradually decreased over time [Figs. 7(c)–7(f)]. TPM injection occurred at 600 s, and approximately 10 min later at 1300–1600 s, the gamma power in PTZ+TPM rats was significantly lower (PTZ+TPM: 38.5% ± 11.9% of the peak value vs PTZ: 59.2% ± 3.0% of peak value; unpaired *t* test with Welch's correction; ^*^p < 0.05; PTZ: n = 3, PTZ + TPM: n = 5) than that in PTZ rats [Fig. 7(d)]. At the 6900–7200 s period, gamma power in both PTZ+TPM and PTZ rats dropped back to near baseline levels [PTZ+TPM: 29.7% ± 6.0% of the peak value vs PTZ: 45.7% ± 20.3% of peak value; Fig. 7(f)].

### F. Neurovascular coupling in 4-AP-induced epileptic seizures

**FIG. 8.**
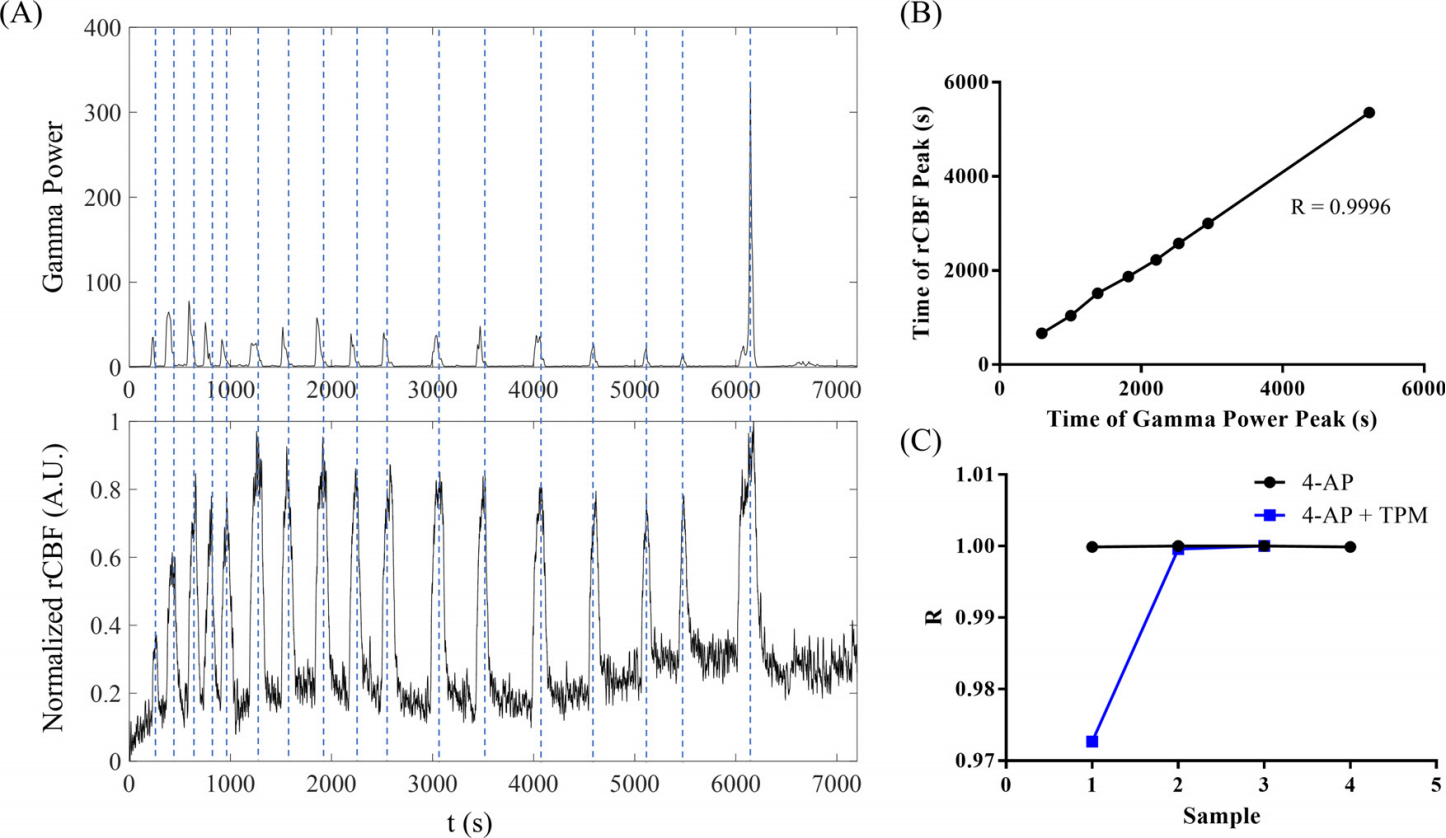
Neurovascular coupling in 4-AP-induced epileptic seizures. (a) An example of gamma power (top panel) and the normalized rCBF (bottom panel) during 4-AP-induced seizure activity. The gamma peaks matched the rCBF peaks over time, demonstrating neurovascular coupling. (b) An example plot of the time of peaks in rCBF vs the time of peaks in gamma power, demonstrating a correlation with an r value of 0.9996. (c) Plot of Pearson correlation coefficients calculated for 4-AP (n = 4) and 4-AP+TPM animals (n = 3). All r values fell between 0.97 and 1, showing strong correlation and neurovascular coupling.

By comparing the peaks in gamma power to rCBF [Fig. 8(a)], we identified neurovascular coupling in 4-AP-induced seizures. The peaks in neural activity aligned with peaks in rCBF. Peaks in gamma power preceded rCBF peaks by 40.5 ± 26.6 s, demonstrating that an increase in blood flow occurs after neural activity. Figure 8(b) shows the time of rCBF peaks plotted against the time of gamma-power peaks in one rat, and the Pearson correlation coefficient (r) was calculated as 0.9996, indicating a strong correlation. r was calculated for all samples [Fig. 8(c); 4-AP: n = 4; 4-AP+TPM: n = 3] and ranged from 0.9727 to 1.0000, indicating a strong correlation.

### G. Spatiotemporal changes in rCBF in 4-AP-induced focal epileptic seizures

**FIG. 9.**
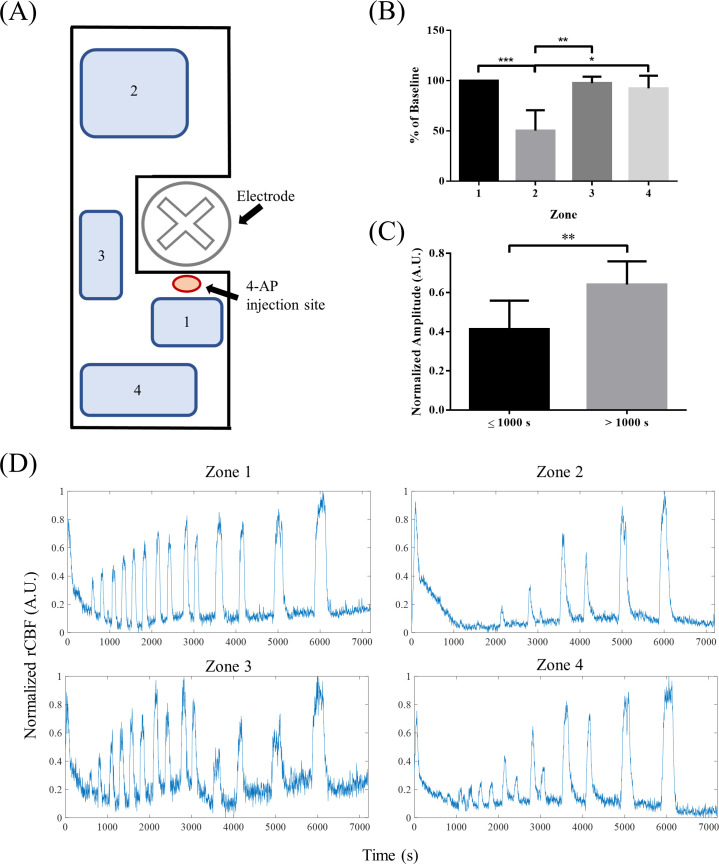
Changes in rCBF in 4-AP-induced focal epileptic seizures. (a) To determine the effect of distance, rCBF was calculated for four different zones in the cranial window with different distances from the 4-AP injection site. (b) The number of peaks normalized to the baseline (zone 1) was significantly lower at the location furthest from the injection site (zone 2) than in any of the other zones (Kruskal–Wallis one-way ANOVA with Dunn's multiple comparisons test; ^*^p < 0.05, ^**^p < 0.01, and ^***^p < 0.005; N = 7). (c) The amplitude of peaks was significantly smaller at the beginning of the seizure (*t* ≤ 1000 s) than later during the seizure (*t* > 1000 s; unpaired t test with Welch's correction; ^**^p < 0.01; N = 7). (d) Example images of change in rCBF over time in zones 1–4.

To determine the effect of distance on rCBF in focal epileptic seizures, rCBF was calculated in four zones varying in distance from the 4-AP injection site. Figure 9(a) shows a representative drawing of the zone locations in the cranial windows shown in Fig. 1. Zone 1 was closest to the injection site, zone 2 was the furthest, and zones 3 and 4 were at a medium distance but in different directions. The numbers of rCBF peaks were compared among the four zones [Fig. 9(b)]. Since the total number of peaks varied in different animals, data were normalized to baseline, which was defined as the number of peaks in zone 1. The number of peaks relative to baseline in zone 2 was significantly lower than that in all the other zones (zone 1: 100.0% ± 0.0%, zone 2: 50.3% ± 20.2%, zone 3: 97.6% ± 6.3%, and zone 4: 92.5% ± 12.4%; Kruskal–Wallis one-way ANOVA with Dunn's multiple comparisons test; ^*^p < 0.05, ^**^p < 0.01, ^***^p < 0.005; and N = 7), demonstrating decreased rCBF fluctuation at distances further from the focal zone. Zones 3 and 4 were not significantly different, demonstrating that there was no difference in rCBF directionality. The amplitudes of rCBF peaks at the beginning of the seizure (≤1000 s) were also compared to those later during the seizure (>1000 s) in Fig. 9(c) (unpaired *t* test with Welch's correction; ^**^p < 0.01; N = 7). Amplitudes at the beginning (0.41 ± 0.15 A.U.) of seizures were significantly smaller than those later during seizures (0.64 ± 0.12 A.U.), demonstrating that fluctuations in rCBF were suppressed at the beginning of the seizure. An example of normalized rCBF in the four different zones is shown in Fig. 9(d).

## III. DISCUSSION

Using our ECoG-LSCI system, we were able to characterize epileptic seizures induced by injection of 4-AP or PTZ. 4-AP and PTZ are widely used in epilepsy research to induce cortical seizures, albeit through different neuronal mechanisms. 4-AP inhibits voltage-gated potassium channels, dramatically increasing the concentration of extracellular potassium. This leads to a decrease in the excitability threshold of neurons, resulting in massive neural firing in the injection zone.^16^ This neural firing rapidly synchronizes, leading to the formation of focal epileptic seizures. In contrast, PTZ is a selective inhibitor of postsynaptic GABA receptors; GABA is the main inhibitory neurotransmitter of the central nervous system. Thus, the epileptogenic effect of PTZ is not based on an increase in excitability but on a decrease in inhibition; these inhibitory neurons, under normal conditions, prevent pathological synchronization and the formation of epileptic seizures. Both the PTZ model and the 4-AP model are widely used for testing antiepileptic drugs.^19^

In the 4-AP model, multiple clusters of high-frequency spike waves were observed via ECoG as high-frequency spikes with increased amplitude. These seizure events corresponded to changes in rCBF observed in LSCI data, indicating neurovascular coupling during epileptic seizures. These changes in CBF may be a contributing factor to seizure-induced neuronal injury and indicate a sharp increase in local blood flow in the area of epileptic seizures. This finding is highly consistent with accumulated data on CBF in epileptic foci.^14^ Notably, TPM induced a decrease in local blood flow, indicating that seizure imposes massive metabolic strain on the cortex, and it is possible that the oxygen demand exceeds supply in the seizure focus. Since there are no data regarding the direct effect of TPM on neurovascular coupling, it is logical to assume that a decrease in neural firing in the area of epileptic seizures reduces the intensity of local metabolism, which leads to a decrease in local blood flow.

Comparison between untreated rats and rats treated with the antiepileptic drug TPM showed decreased ECoG signal amplitudes of the spike wave clusters induced by 4-AP. Decreasing the amplitude of abnormal neural activity may limit neuronal damage. In PTZ-induced seizures, TPM treatment decreased ECoG signal power in the gamma spectral band. The effect of TPM on electrophysiological signals is unclear. Studies of TPM administration have shown no change in EEG signals in healthy volunteers,^20^ while epilepsy patients had changes in the delta and theta bands but not in the gamma band.^21^ Studies have also shown slowing of EEG signals with TPM administration.^22,23^ However, the high-frequency gamma band was associated with the preseizure state in a study using the PTZ model of epilepsy. Other studies have suggested that high-frequency oscillations over time could increase seizure probability. Because TPM was given after seizure induction in our study instead of before, it is unclear whether the suppression of high-frequency oscillation can suppress seizure activity after seizure has begun.

Using LSCI, researchers have been able to demonstrate changes in CBF during PTZ-induced absence seizures in freely behaving rats, which are commonly used as an animal model in epilepsy research. PTZ causes absence-like seizures originating in the thalamus and spreading to the cortex. The use of LSCI allowed for the detection of CBF changes during these seizures, as previously shown using BOLD fMRI, diffuse optical tomography (DOT), and photoacoustic tomography (PAT).^24^ The ability to monitor CBF changes during seizures using LSCI provides a valuable tool for better understanding the underlying mechanisms of epilepsy and potentially developing new treatment strategies.

By integrating ECoG with LSCI, we observed neurovascular coupling in 4-AP-induced seizures. Similarly, other studies measuring total hemoglobin concentration, CBF, or blood vessel diameter during 4-AP-induced seizures have reported changes corresponding to electrical activity.^25–27^ Our finding that neural activity preceded the vascular response by 40.5 ± 26.6 s is surprising because increases in CBF due to neural stimulation last only a couple of seconds.^28^ One explanation is that we measured the time at which peak CBF occurred instead of the time at which CBF first began to increase above baseline. An increase in CBF provides metabolic support for increased neural activity. Since neural stimulation has a shorter timescale than epileptic seizures, it is reasonable to observe a greater lag to the peak value of CBF. Nevertheless, peak neural activity preceded a peak increase in CBF. We also found that the peak amplitudes at the beginning of the seizure were significantly lower than those later during the seizure. This difference is possibly due to the failure of inhibitory mechanisms after 1000 s. Our data also showed that changes in CBF in regions farther away from the 4-AP injection site lacked some of the initial peaks seen in regions closer to the injection site. This is possibly due to the partial suppression of seizures in closer regions and full suppression of seizures in farther regions. Eventually, the inhibitory mechanisms fail, and the seizures fully propagate into distant regions. Other studies^29,30^ have also found regions of inhibition around the epileptic focus, possibly from inhibitory interneurons.^31^ Further work is required to clarify the mechanisms underlying inhibition and inhibition failure. In addition, it is unclear whether the same events observed in 4-AP-induced seizures in rats also occur in epilepsy in humans.

It seems logical to assume that TPM inhibits epileptic activity by protecting the blood–brain barrier (BBB) in the epileptic focus from developing pathology. This assumption is supported by data on the protective effect of TPM on the functions of the BBB.^32^ The relationship of the BBB with epileptic pathologies has been carefully studied in recent years.^33^ It has been shown that local or general disruption of BBB function is associated with an increase in the level of glutamate, which inevitably occurs in the development of epileptic seizures. In turn, a disturbed BBB will reduce the self-regulation ability aimed at suppressing epileptic seizures.

The action of TPM can be summarized, with slight simplification, as follows: (I) inhibition of voltage-dependent sodium channels, (II) potentiation of GABA-mediated transmission, (III) inhibition of non-N-methyl-D-aspartate glutamate receptors, (IV) negative modulation of L-type calcium channels, and (V) inhibition of carbonic anhydrase isoenzymes.^34^ Thus, the action of TPM is complicated. It is natural that TPM has a different effect on seizures caused by 4-AP and PTZ. The most likely scenario is that 4-AP-induced focal seizures not only increase the permeability of the BBB but also come accompanied by an increase in the potassium concentration in the extracellular space. This scenario would largely account for the difference between the 4-AP- and PTZ-induced seizures that we observed.

Based on our imaging and electrophysiological data, we can conclude that BBB disruption may increase the likelihood of an epileptic seizure. The converse is also true: local destruction of the BBB caused by an epileptic seizure can increase epileptic activity. An epileptogen causes disruption of neural circuit function during a trigger event, and disruption of the BBB may determine neural circuit function during epileptogenesis. This concept of the role of the BBB in epileptogenesis was put forward earlier, and our data probably confirm it.^35^ Data obtained using *in vivo* optical imaging, including LSCI, are largely determined by the functions of the BBB, which also confirms its role in the mechanism underlying the antiepileptic action of TPM.

In future human applications of ECoG-LSCI, it will be necessary to use a camera that can capture a larger ROI and integrate this camera with scalp-contacting headgear onto which the ECoG electrode is placed. Optical fibers can be used instead of optical mirrors for increased portability, which will allow for longitudinal monitoring of epileptic seizures.

The current clinical applications of this technology consist mainly of monitoring CBF during various neurosurgical interventions, such as aneurysm clipping, vessel bypass, and tumor removal^36^ as well as localization of the sensory, motor, and speech centers in the cerebral cortex.^36^ Indocyanine green angiography (ICGA), Doppler sonography, and transfemoral digital subtraction angiography (DSA) are commonly used methods, but each has its drawbacks. ICGA requires an intravenous injection of a fluorescent indocyanine green (ICG) bolus and cannot provide continuous imaging. DSA requires multiple time-controlled x-rays with intra-arterial contrast medium injection, while Doppler ultrasound is limited to measuring in single locations and requires mechanical contact with the vessel. DSA is also invasive and time consuming. Optical imaging of intrinsic signal (IOSI) is based on the difference in the optical properties of oxy- and deoxyhemoglobin and does not require additional chemicals, but it usually takes additional time.^37^

LSCI of cerebral blood flow is a promising method that is intensively used in functional brain imaging studies. LSCI produces real-time blood flow maps noninvasively and without any chemical probes.^17,38^ This is important, as epileptic seizures often involve changes in CBF in the focus of the epileptic activity. LSCI is also a noninvasive method that enables research to be conducted without any instrument coming in direct contact with the brain, reducing the risk of complications and increasing safety for the patient. Currently, LSCI is widely used in epilepsy research, including studies of the mechanisms of epileptic seizures and the effectiveness of antiepileptic drugs in reducing seizures. LSCI can also be used to differentiate epileptic seizures from other types of seizures. Thus, LSCI is a valuable tool for epilepsy research, including drug discovery, and can aid in understanding the mechanisms of epilepsy and developing more effective treatments.

LSCI has been repeatedly attempted for functional human brain mapping. In a pilot clinical study, LSCI was used in real time during a neurosurgical operation.^39^ Imaging was performed before and after therapeutic coagulation on the open brain, while EEG recording was carried out simultaneously. The study demonstrated the potential of LSCI to produce a CBF map with high spatial resolution. Over the years, the technique of using LSCI during neurosurgical intervention has been significantly improved due to the development of multiple-exposure speckle imaging (MESI).^36^ MESI has been found to provide extremely high sensitivity to cerebral blood flow,^36^ and its application in a group of patients demonstrated that intraoperative MESI can be performed with high quantitative accuracy and sensitivity for monitoring cerebral blood flow.^36^

ECoG-LSCI of the whole brain would require a penetration depth of several centimeters for the laser light. However, the 660-nm-wavelength light used in this study has a penetration depth of less than 1 mm. Due to this limitation, the acquired images represent the optical absorption distribution several millimeters under the cortical surface rather than the whole brain. Near-infrared (NIR) light can be used to increase the penetration depth. For example, 800 nm wavelength light can penetrate as deep as 2 mm in tissue. Employing a cutting-edge photoacoustic (PA) system can also allow whole brain imaging in a preclinical model. Notably, while blood-tissue contrast is lower at NIR wavelengths than at 532 nm, it is still detectable using the PA system.^40^ In addition, NIR contrast agents such as ICG can be used to further enhance contrast for PA epilepsy imaging.

The mechanism of action of TPM remains unclear, but some researchers have hypothesized that it acts on voltage-gated sodium channels.^41^ Thus, it has also been suggested to act on voltage-gated calcium channels. Other candidates for TPM targets are GABA_A_ receptors, AMPA receptors, and kainate receptors.^41^ In addition, TPM increased the frequency of GABA-mediated chloride channel opening and increased potassium conductance.^42^ Many researchers believe that the antiepileptic effect of TPM is due mainly to its influence on sodium channels.^42^ TPM (25 mg/kg) was demonstrated to be unable to prevent the epileptiform EEG activity induced by 4-AP.^43^ In our study, TPM exhibited antiepileptic activity via potassium channel inhibition.

However, the addition of 25–50 *μ*M TPM reduced the frequency of epileptiform bursts in a study using a hippocampal slice model with 4-AP-induced epilepsy. The researchers concluded that TPM modulates Na^+^-independent Cl-/HCO^3−^ exchange. Neuronal intracellular pH (pHi) is most likely lowered through a combined effect on Na^+^-independent Cl-/HCO^3−^ exchange and Ca^2+^, and such a decrease in pHi may contribute to the antiepileptic effect of TPM.^44–46^ These data are consistent with those of another study in which TPM reduced the intrinsic optical signal (IOS) induced by 4-AP in living brain slices and their spread to distant areas.^47^ Additionally, in hippocampal slices, TPM was shown to significantly decrease sustained repetitive firing in pyramidal neurons.^48^ It is logical to assume that TPM inhibits synaptic conductance responsible for the transmission of synchronized epileptiform activity.^48^ However, it is likely that the action of TPM *in vivo* is not as simple. The induction, spread, and decay of seizures in the cortex *in vivo* is associated with glia and local blood flow.^19^ Local blood flow is usually the main contributor to the formation of optical correlates of epileptic seizures.^17^ Thus, our data represent a step toward elucidating the mechanisms of action of TPM on neurovascular coupling as well as on neural circuits.

The mechanism underlying the antiepileptic effect of TPM in PTZ-induced epileptic seizures is not entirely clear. Synergistic effects of combinations of TPM and lamotrigine in the PTZ model have been demonstrated, but neither TPM nor lamotrigine alone exerts a sufficiently strong antiepileptic effect to prevent PTZ-induced seizures.^49^ Additionally, long-term use of TPM decreased cognitive function in an experimental model of epilepsy.^50^ This decrease is probably due to TPM-induced selective inhibition of voltage-gated sodium channels, stabilization of presynaptic membranes, and inhibition of the release of glutamate and aspartate from presynaptic neurons. There is no evidence that TPM affects serotonin, norepinephrine, or dopamine transmission.^51^ It is possible that lamotrigine acts on voltage-gated calcium channels, which would contribute to its wide spectrum of activity.

In a human study, TPM was shown to drastically increase brain concentrations of GABA in healthy individuals.^52,53^ Regarding its molecular mechanism, TPM interacts with the alpha-1 subunit of the GABA receptor and is a GABA agonist. At nonbenzodiazepine receptors in brain neurons, TPM stimulates the activity of GABA_A_ receptors and reduces the activity of glutamate AMPA and kainate receptors.^54^

Currently, TPM is widely used in the clinic as an antileptic drug, either alone or in combination with other medicines.^41^ The mechanisms underlying the antiseizure effect of TPM have been studied by different methods in the last few decades. Most likely, its effect involves multiple mechanisms, including (but not limited to) the blockade of voltage-gated Na^+^ channels, inhibition of voltage-gated Ca^2+^ channels, inhibition of glutamate synapses, and enhancement of GABA neurotransmission.^41^

In summary, we can confidently say that there is a need for a method for noninvasive and reliable monitoring of CBF both in the clinic and in preclinical epileptic studies. LSCI, which provides fast and reliable visualization of CBF in a cortical area, very successfully meets the profile for such a methodology. In our study, we used LSCI to study local CBF in two epilepsy models to test the antiepileptic drug TPM. ECoG was used to verify the duration of epileptic seizures and to compare ictal and interictal data. In future research, the ECoG-LSCI system can be applied to other animal models of epilepsy. Genetically modified animals can be used to understand the effect of specific genetic defects on the neurovascular aspect of cortical epileptic seizures. Promising antiepileptic drugs can also be tested, including selective gap junction inhibitors. The combination of LSCI and ECoG may provide new information about the role of the astrocytic syncytium in the development of epileptic seizures and the possible use of its gap junctions as a therapeutic target. Currently, LSCI is widely used in epilepsy research, including studies of the mechanisms of epileptic seizures and the effectiveness of antiepileptic drugs in reducing seizures. LSCI can also be used to differentiate epileptic seizures from other types of seizures. Thus, LSCI is a valuable tool for epilepsy research, including drug discovery, and can aid in the understanding of its mechanisms and the development of more effective treatments.

## IV. CONCLUSION

We tested a rat model of 4-AP-induced epileptic seizures and were able to characterize these seizures using an in-house ECoG-LSCI system. We observed distinct differences in the ECoG signal between TPM-treated and untreated animals in 4-AP-induced seizures. Treatment significantly decreased the signal amplitude (4-AP+TPM: 0.61 ± 0.13 mV vs 4-AP: 1.08 ± 0.19 mV). Consistent with these findings, other studies have reported that TPM treatment induces changes in EEG recordings during status epilepticus. This demonstrates that our ECoG-LSCI system can be used to evaluate the effectiveness of different treatments. We also tested another animal model of epileptic seizures using PTZ. Gamma-band power analysis was used to characterize seizure patterns and assess the treatment efficacy of TPM. Comparison of TPM-treated vs untreated PTZ-induced seizures showed significantly decreased gamma power (PTZ+TPM: 38.5% ± 11.9% of the peak value vs PTZ: 59.2% ± 3.0% of peak value) in treated animals at approximately 10 min after TPM injection. This demonstrates that our ECoG-LSCI system can be applied to different animal models of epilepsy. Previously, it was suggested that the antiepileptic properties of TPM could be explained by a decrease in pHi in a model of 4-AP-induced epileptic seizures.^44^ Our data indirectly support this hypothesis. We successfully demonstrate *in vivo* epilepsy mapping with high spatial and temporal resolution in an animal model. Simultaneously, we employed electrophysiological recording to document the dynamics of epileptic seizures. A combination of these methods was successfully used to monitor the antiepileptic effect of a particular drug. The main advantage of this combination of optical and electrophysiological methods is the independent acquisition of data on neuronal activity and neurovascular coupling in the epileptic focus. The mechanism of action of TPM on epileptiform activity remains unclear. PTZ, a GABA receptor antagonist, was used to create a common chemically induced seizure model; in contrast to the 4-AP model, PTZ-induced seizures represent generalized seizures (not focal seizures). It seems reasonable to assume that its antiepileptic activity may be associated with an effect on the alpha-1 subunit of the GABA receptor, but this hypothesis requires additional research. Visualization of changes in local blood flow and oxygenation using optical methods in animal models of epilepsy has become widespread. However, the use of LSCI remains relatively limited. We demonstrated the use of an LSCI system in combination with ECoG recording. Changes in rCBF can be a clearer indicator of seizure activity than ECoG in cases such as when seizure activity was partially suppressed by TPM, suggesting that further use of LSCI in tandem with electrophysiological methods will be extremely useful in the search for new antiepileptic drugs.

## V. METHODS

### A. Electrocorticography and laser speckle contrast imaging of epileptic seizures

A schematic of our ECoG-LSCI system is shown in Fig. 1. A 660 nm laser (RM-CW04–100, Unice E-O Service Inc., Taoyuan, Taiwan) was used to illuminate the cortical surface for LSCI imaging. The light path included a plano–convex lens (f = 75 mm, LA1608-A, Thorlabs Inc., Newton, NJ, USA) such that the illumination area was approximately 40 × 30 mm. Laser light scattered and reflected from the cortical surface was imaged using a 16-bit charge-coupled device (CCD) camera (DR2–08S2M/C-EX-CS, Point Gray Research Inc., Richmond, BC, Canada) with an adjustable magnification lens (0.3–1×, f/4.5 max) and 2× extender. A linear polarizer was added to eliminate scattering. LSCI images (1032 × 1384 pixels) were acquired at 2 fps. A custom LabVIEW (National Instruments, Austin, TX, USA) program was written to control the LSCI system, while laser speckle contrast analysis was implemented using MATLAB (MathWorks Inc., Natick, MA, USA). The movement of erythrocytes, or red blood cells (RBCs), near the cortical surface causes interference in the scattered and reflected laser light, which generates speckle patterns.^55^ Changes in the speckle pattern can be quantified to measure changes in blood flow.^13^

ECoG signals were collected from electrodes implanted in the rat skull and processed with a 128-channel Neural Signal Processor (Blackrock Microsystems, Salt Lake City, UT, USA). The ECoG signals were recorded through the head-stage amplifier (gain of 2) and filtered by a bandpass filter from 0.5 to 150 Hz. The signals were then digitized at a sampling rate of 10 kHz.

### B. Animal model of 4-AP-induced epileptic seizures

Animal experiments were performed according to guidelines from the Institutional Animal Care and Use Committee (approval number: IACUC-NHRI-108098). Sprague–Dawley rats (BioLASCO Taiwan Co., Ltd., Taiwan) weighing 250–500 g were anesthetized with 1%–3% isoflurane (Panion & BF Biotech Inc., Taiwan) in oxygen from craniotomy to the end of seizure recording. For craniotomy, animals were secured on a stereotaxic frame (Stoelting Co., Wood Dale, IL, USA), and an incision was made in the skin to expose the skull. Using the bregma as a landmark, a cranial window with the dimensions ±3.0 mm anterior–posterior (A–P) and 0.0, +4.0 mm medial–lateral (M–L) was made using a high-speed drill. For ECoG, an epidural electrode was secured at +1.0 mm A–P and +4.0 mm M–L for monitoring seizure activity, and a reference electrode was secured at +4.0 mm M–L to the lambda landmark (Fig. 1).

For induction of epileptic seizures, 0.5 *μ*l of 30 mM 4-AP (Sigma-Aldrich, St. Louis, MO, USA) solution was injected into the cortex at −1.0 mm A–P and +4.0 mm M–L, approximately 0.5 mm below the dura mater. A 10-*μ*l glass syringe (Hamilton Co., Reno, NV, USA) was used for accurate injection. A schematic of the data collection is shown in Fig. 2. With the ECoG-LSCI system, data were recorded for 30 min before 4-AP injection to obtain baseline values and for 2 h after 4-AP injection to monitor 4-AP-induced epileptic seizures.

### C. Animal model of PTZ-induced epileptic seizures

After craniotomy as described in Sec. V B, rats were administered an intraperitoneal (i.p.) injection of PTZ (Sigma-Aldrich, St. Louis, MO, USA) at a dose of 30 mg/kg. The data collection schematic is shown in Fig. 2. A 30 min of baseline values and 2 h of seizure data were collected using the ECoG-LSCI system.

### D. Treatment with topiramate

Control rats were divided into two groups, one with 4-AP induction of seizures (group 1) and the other with PTZ induction of seizures (group 3). In experimental group 2, TPM (30 mg/kg; Sigma-Aldrich, St. Louis, MO, USA) was injected i.p. at 10 min before 4-AP seizure induction. In experimental group 4, TPM (30 mg/kg) was injected i.p. at 10 min after seizure induction with PTZ. Data collection for TPM-treated rats followed the same schematic as control rats, as shown in Fig. 2.

### E. Data analysis for LSCI

The laser speckle contrast (*K*)^56^ was computed from speckle images in windows of 5 × 5 pixels as follows:

$$K=\frac{\sigma }{\left\langle I\right\rangle },$$ (1)

where σ is the standard deviation and $\left\langle I\right\rangle$ is the local mean of the speckle intensity pattern.

The speckle contrast is then related to CBF as follows:

$$\mathrm{CBF}\propto \frac{1}{TK^{2}},$$ (2)

where *T* is the exposure time. With constant *T*, we can then calculate regional CBF (rCBF) to be the inverse of $K^{2}$.

### F. Data analysis

ECoG-LSCI data were analyzed using MATLAB software. An example of a 4-AP-induced ECoG seizure pattern is shown in Fig. 3(a). The time of seizure onset (t_0_), number of spike wave clusters, average cluster amplitude, average cluster duration, and total seizure duration were quantified. An unpaired *t* test with Welch's correction was used to compare TPM-treated animals to untreated animals in Prism 6 (GraphPad Software, Inc., San Diego, CA, USA).

Gamma (80–120 Hz) spectral-band power was calculated using a 30-s moving window with 80% overlap.^57,58^ To observe neurovascular coupling in 4-AP-induced seizures, gamma power was plotted and compared to rCBF values. The times at which peaks occurred were identified, and the Pearson correlation coefficient (r) was calculated to determine whether there was a correlation between gamma power and rCBF. For PTZ-induced seizures, six different 300-s intervals were selected for comparison of average gamma power between TPM-treated and untreated animals. To normalize data collected from different animals, gamma power was transformed to the percent of peak value. An unpaired t test with Welch's correction was used for statistical analysis.

To investigate the effect of distance on seizure-induced changes in rCBF in the 4-AP model of focal epilepsy, the normalized number of peaks in four different zones in the cranial window were compared using a Kruskal–Wallis one-way analysis of variance (ANOVA) and Dunn's multiple comparisons test. The average peak amplitudes before and after 1000 s were also compared using an unpaired t test with Welch's correction to analyze the effect of time on the rCBF response. Peaks in rCBF at the beginning (t = 0) were excluded from amplitude analysis since there was no corresponding ECoG activity; these peaks were assumed to be an artifact from 4-AP injection.

## SUPPLEMENTARY MATERIAL

See the supplementary material for movies of the change in rCBF measured using LSCI during 4-AP induction of epileptic seizure in a control (Movie 1) and a TPM-treated rat (Movie 2).

## Data Availability

The data that support the findings of this study are available from the corresponding author upon reasonable request.
